# EGFR is a potential dual molecular target for cancer and Alzheimer’s disease

**DOI:** 10.3389/fphar.2023.1238639

**Published:** 2023-08-02

**Authors:** Hee-Jeong Choi, Yoo Joo Jeong, Jieun Kim, Hyang-Sook Hoe

**Affiliations:** ^1^ Department of Neural Development and Disease, Korea Brain Research Institute (KBRI), Daegu, Republic of Korea; ^2^ Department of Brain and Cognitive Sciences, Daegu Gyeongbuk Institute of Science and Technology, Daegu, Republic of Korea; ^3^ Department of Bio-Health Technology, College of Biomedical Science, Kangwon National University, Chuncheon, Republic of Korea

**Keywords:** Alzheimer’s disease, EGFR, EGFR inhibitor, cancer, Aβ, learning and memory

## Abstract

Many researchers are attempting to identify drugs that can be repurposed as effective therapies for Alzheimer’s disease (AD). Several recent studies have highlighted epidermal growth factor receptor (EGFR) inhibitors approved for use as anti-cancer drugs as potential candidates for repurposing as AD therapeutics. In cancer, EGFR inhibitors target cell proliferation and angiogenesis, and studies in AD mouse models have shown that EGFR inhibitors can attenuate amyloid-beta (Aβ) pathology and improve cognitive function. In this review, we discuss the different functions of EGFR in cancer and AD and the potential of EGFR as a dual molecular target for AD diseases. In addition, we describe the effects of anti-cancer EGFR tyrosine kinase inhibitors (TKIs) on AD pathology and their prospects as therapeutic interventions for AD. By summarizing the physiological functions of EGFR in cancer and AD, this review emphasizes the significance of EGFR as an important molecular target for these diseases.

## 1 Introduction

Heredity and aging are common risk factors for cancer and Alzheimer’s disease (AD), which are the leading causes of death worldwide (White et al., 2014; Guerreiro and Bras, 2015; Majd et al., 2019). Numerous anti-cancer therapies are available, but therapeutic drugs for AD are scarce. AD is the most common neurodegenerative disease characterized by amyloid and tau protein aggregation and cognitive decline (Ates et al., 2016). Amyloid and tau protein aggregates are not only pathophysiological biomarkers of AD, but also cause neuronal loss, synapse destruction, and neuroinflammation (Serrano-Pozo et al., 2011; Krstic and Knuesel, 2013). Other potential mechanisms of AD progression are oxidative stress and epigenetic dysfunction.

Several studies have found an inverse association between cancer and AD, but others have argued for a parallel relationship (Majd et al., 2019; Nudelman et al., 2019; Branigan et al., 2021; Zhang et al., 2022). Although the underlying mechanism of the relationship between cancer and AD has not been thoroughly investigated, the diseases share hallmarks and risk factors. Risk factors for both cancer and AD include aging, smoking, obesity, and type 2 diabetes (Cannata et al., 2010; Cataldo et al., 2010; Mayeux and Stern, 2012; White et al., 2014; Emmerzaal et al., 2015). Strikingly, cell-cycle entry, which is required for cancer pathogenesis, is high in patients with AD (Majd et al., 2019). At the cellular level, the pathogenesis of AD and cancer both involve the phosphoinositide 3-kinase/protein kinase B/mammalian target of rapamycin (PI3K/AKT/mTOR) signaling pathway, which regulates cell proliferation, metabolism, growth, and autophagy (Pei and Hugon, 2008; Morgan et al., 2009; Advani, 2010; Talbot et al., 2012; Fumarola et al., 2014; Porta et al., 2014). Abnormal growth suppressor evasion is also observed in both cancer and AD. Specifically, cell growth and division in cancer often occur through inactivating mutations of tumor suppressors such as retinoblastoma transcriptional corepressor 1 (RB1) and TP53 (Hanahan and Weinberg, 2011; Fischer et al., 2016; Robinson et al., 2017). In patients with AD, levels of p27, a critical negative cell cycle regulator, are significantly reduced (Ogawa et al., 2003; Munoz et al., 2008). The systemic dysregulation of the cell cycle in both cancer and AD supports a correlation between these two diseases. Angiogenesis, cell adhesion inhibition, and inflammation are also shared by cancer and AD (Nudelman et al., 2019). Therefore, verifying the commonalities between cancer and AD might contribute to the development of effective therapeutic strategies.

A genome-wide association study found a significant positive genetic correlation between cancer and AD, implying that the pathophysiology of cancer and AD share common genetic variants (Feng et al., 2017). Specifically, super-enhancer, a broad enhancer domain affecting cell type identification and function, exhibits a significant positive genetic correlation with cancer and AD, indicating a potential role of gene expression regulation in the common genetic etiologies of cancer and AD (Feng et al., 2017; Zhao et al., 2022). Several genes [e.g., epidermal growth factor receptor (EGFR) and amyloid precursor protein (APP)] are associated with both cancer and AD, and we and others have recently found that anti-cancer drugs can penetrate the blood-brain barrier (BBB) and modulate AD pathology (Ryu and McLarnon, 2008; Cramer et al., 2012). Specifically, inhibitors of EGFR and other tyrosine kinases, which are multitarget enzymes, may have practical value for treating cancer and AD (Mansour et al., 2021c). This review provides insights into the potential roles of EGFR and EGFR inhibitors in cancer and AD and related therapeutic strategies.

## 2 EGFR

EGFR is a cell surface growth factor receptor that regulates cell proliferation, differentiation, and survival (Yewale et al., 2013). EGFR was the first receptor tyrosine kinase (RTK) to be discovered and is a member of the ErbB family of RTKs (Wong and Guillaud, 2004; Roskoski, 2019). The EGFR gene contains 31 exons and encodes a 170-kDa transmembrane glycoprotein (Mitsudomi and Yatabe, 2010; Sabbah et al., 2020). EGFR is stimulated by ligands such as epidermal growth factor (EGF) and transforming growth factor-alpha (TGF-α) (Purba et al., 2017). Upon binding to the extracellular domain of EGFR, these ligands induce conformational changes in the receptor that facilitate the formation of receptor dimers or oligomers (Schlessinger, 2002). EGFR dimerization triggers the activation of its intrinsic tyrosine kinase activity and subsequent autophosphorylation of several tyrosine residues in the EGFR C-terminal domain (Yu et al., 2002). These phosphorylated tyrosine residues serve as docking sites for various signaling molecules and initiate canonical EGFR signaling pathways (Lemmon and Schlessinger, 2010). Although numerous reviews have discussed EGFR as a target in cancer, inflammatory diseases, and monogenic diseases, the regulation of EGFR expression or signaling as a multi-disease target requires further investigation.

## 3 EGFR in various cancers

EGFR plays an essential physiological role in regulating the development of epithelial tissue and homeostasis and hence is also linked to tumorigenesis, including lung cancer, breast cancer, and glioblastoma (Sigismund et al., 2018). EGFR is a critical modulator and a target for developing novel therapeutic strategies in various cancers. EGFR signaling modulates cancer cell proliferation through several metabolic processes (Sigismund et al., 2018). For example, Srivatsa et al. (2017) found that EGFR-expressing myeloid cells are abundant in the colorectal tumor stroma, indicating that EGFR in tumor-associated myeloid cells may be a diagnostic biomarker for colorectal cancer (CRC). CRISPR/Cas9-mediated elimination of EGFR significantly inhibits tumor cell growth and activates the mitogen-activated protein kinase (MAPK) (p-ERK1/2) pathway (Liu et al., 2020). Moreover, Song et al. (2020) identified upregulation of EGFR and phosphorylated signal transducer and activator of transcription 3 (p-STAT3) in breast cancer tissues.

The EGFR pathway is a widely recognized oncogenic pathway for non-small cell lung cancer (NSCLC), which represents approximately 75% of lung cancers (Bethune et al., 2010; Hsu et al., 2019). The EGFR pathway regulates the Bax/B-cell lymphoma 2 (Bcl-2) cascade, which is associated with apoptosis in NSCLC (Alam et al., 2022). Interestingly, a study identified EGFR overexpression or mutations in intracellular EGFR in 43%–89% of NSCLC cases (Gupta et al., 2009). Exon 19 deletion and L858R point mutation are the most frequent EGFR mutations in NSCLC (Khaddour et al., 2021). Activating somatic mutations in exons 18–21 of EGFR in NSCLC can continuously activate the EGFR kinase domain regardless of ligand binding and result in sustained downstream signaling. Several studies have reported that EGFR expression is increased by 40%–89% in NSCLC (Lu et al., 2001; Lynch et al., 2004; Al Olayan et al., 2012). Shao et al. (2022) found that upregulated EGFR signaling induces increased levels of the membrane-bound complement regulatory proteins (mCRPs) CD55 and CD59, thereby promoting tumor immune evasion in lung cancer cells (CD8^+^T). In addition, Ohsaki et al. (2000) observed shorter survival of NSCLC patients with EGFR overexpression. Selvaggi et al. (2004) found that EGFR expression is significantly increased in stage III of NSCLC, implying that EGFR levels are a potential prognostic factor. Merrick et al. (2006) observed a significantly positive relationship between EGFR expression and the progression of bronchial dysplasia, a precursor of lung carcinoma, indicating a role of EGFR upregulation in lung cancer development and progression. Yang et al. (2015) reported that EGFR mutation-mediated lung cancer is associated with downregulation of cluster of differentiation 82 (CD82), which promotes EGFR expression. Shien et al. (2012) discovered that EGFR silencing by siRNA significantly reduces the cell viability of EGFR-mutant cell lines (PC-9, HCC827, NCI-H820, and NCI-1975), further supporting EGFR as a promising therapeutic target in NSCLC. This critical role of EGFR upregulation in the development, progression, and longevity of lung cancer has led to the development of drugs that control EGFR activity and expression.

## 4 EGFR in Alzheimer’s disease (AD)

**FIGURE 1:**
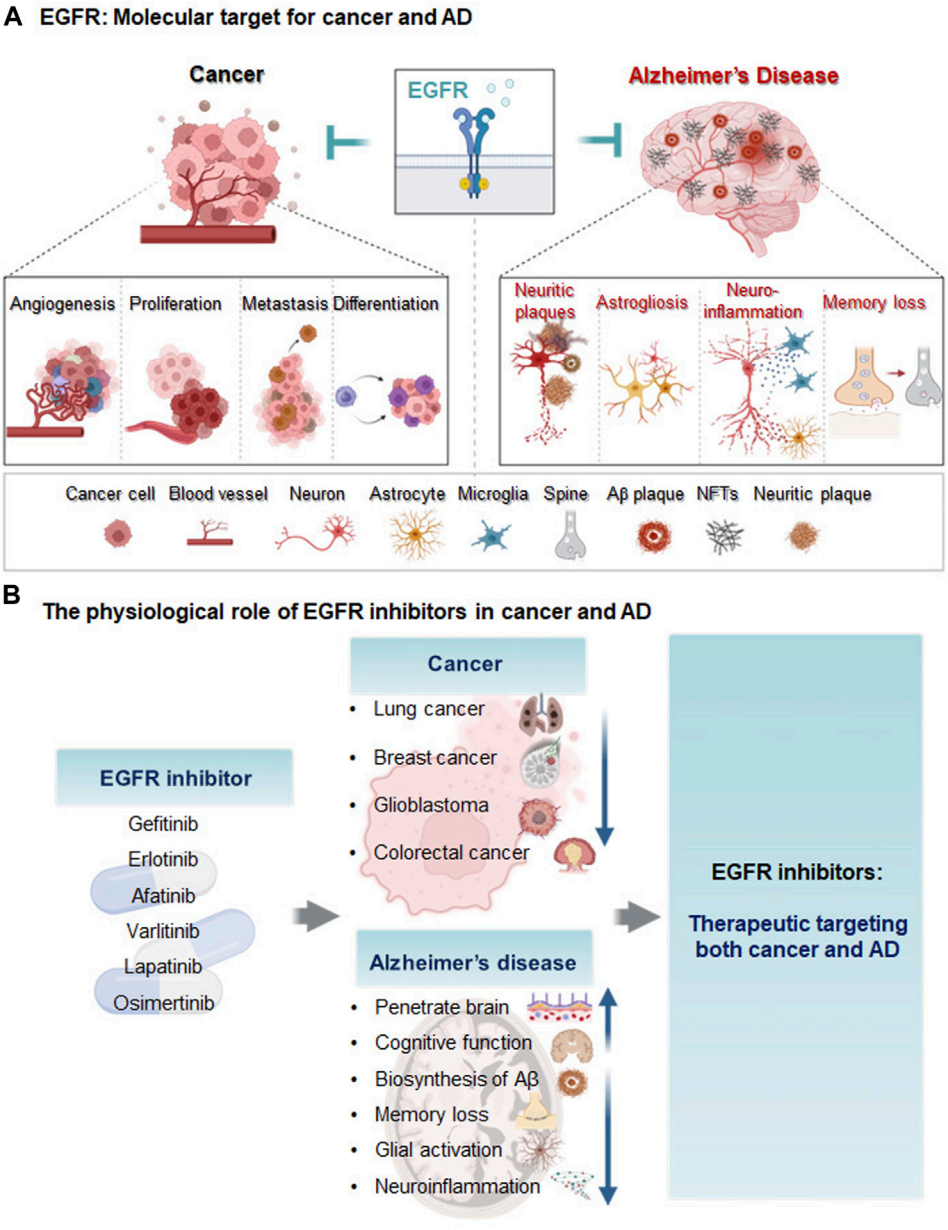
Diagram of EGFR as a molecular target and the effects of EGFR inhibitors on cancer and AD. **(A)** Epidermal growth factor receptor (EGFR) is a transmembrane protein receptor for the epidermal growth factor family that regulates cell growth and proliferation. In cancer, EGFR upregulation increases metastasis, angiogenesis, cancer cell proliferation, differentiation, and cancer viability. In animal models of AD and/or AD patients, EGFR levels are increased, leading to memory loss, astrogliosis, neuroinflammation and Aβ plaque formation. **(B)** The EGFR inhibitors gefitinib, afatinib, varlitinib, erlotinib, osimertinib, and lapatinib are anti-cancer drugs targeting EGFR. These EGFR inhibitors reduce AD pathology and improve cognitive function and thus may be potential therapeutic agents for cancer and AD.

The general functions of EGFR in the central nervous system (CNS) include neural stem cell pool maintenance, astrocyte differentiation and maturation, oligodendrocyte maturation, and neurite outgrowth (Romano and Bucci, 2020). EGFR isoforms are expressed in neurons in the hippocampus, cerebellum, and cerebral cortex (Wong and Guillaud, 2004). Several recent studies have demonstrated that EGFR and its related signaling pathways that it mediates are crucial targets for modulating AD pathology. For instance, Wang et al. (2013) found that EGFR activation (p-EGFR/EGFR) is significantly increased in 8-month-old APP/PS1 mice (a model of AD) compared with wild-type mice. Excessive EGFR expression induces memory impairment in Aβ-overexpressing *Drosophila* (Prüßing et al., 2013). More importantly, dual overexpression of EGFR and Aβ_42_ synergistically promotes memory loss, implying that EGFR is upregulated in AD (Chiang et al., 2010). Notably, EGFR upregulation was recently shown to induce Aβ_42_ neurotoxicity and neuroinflammation and activate astrocytes (Ozbeyli et al., 2017; Chen et al., 2019b). AD patients exhibit neuritic plaques with EGFR expression in the cerebral cortex and hippocampus (Birecree et al., 1988). However, EGF treatment does not alter EGFR or Aβ levels in the brain and prevents cognitive dysfunction in E4FAD mice (Thomas et al., 2016). Taken together, the literature suggests that inhibition of EGFR modulates Aβ plaque accumulation, neuroinflammation, and cognitive function in mouse models of AD. Although there are conflicting reports regarding the role of EGFR in Aβ pathology, there is strong evidence that EGFR is a dual molecular target for cancer and AD (Figure 1A).

## 5 Therapeutic applications of EGFR inhibitors

Despite substantial investments of resources and time in identifying new drugs for AD, clinical trials have produced disappointing results. Thus, the effects of EGFR on Aβ, neuroinflammation, and cognitive function have spurred growing interest in the potential repurposing of EGFR inhibitors used as anti-cancer drugs for the treatment of AD (Mansour et al., 2021c). The molecular mechanisms of EGFR in cancer and AD are also being investigated to develop disease-modifying drugs. Chen et al. (2019b) found that oxygen-glucose deprivation (OGD) increases EGFR phosphorylation and triggers downstream protein kinase B (AKT) and extracellular signal-regulated kinase (ERK) signaling pathways in primary cultured astrocytes and CTX-TNA2 cells. Characterization of EGFR signaling pathways and downstream cascades may reveal promising strategies for utilizing tyrosine kinase inhibitors (TKIs) as disease-modifying therapies in cancer and AD. In addition, EGFR TKIs have greater BBB penetration potential than most intravenous chemotherapies (Ahluwalia et al., 2018). A recent high-performance liquid chromatography (HPLC) analysis showed that ibrutinib can cross the BBB in WT mice (Lee et al., 2021a), and gefitinib, erlotinib, afatinib, varlitinib, lapatinib, and osimertinib are all known to cross the BBB (Lin et al., 2008; Babu et al., 2015; Mansour et al., 2021b; Colclough et al., 2021). Colclough et al. (2021) compared the BBB permeability of EGFR TKIs and found that osimertinib has the highest BBB penetration, with a Kpuu of 0.21, followed by Kpuu values of 0.084 for erlotinib, 0.0092 for gefitinib, and 0.0046 for afatinib. The low BBB permeability of erlotinib and gefitinib means that these drugs do not exhibit significant or persistent effects in the brain (Ahluwalia et al., 2018). Although the abilities of lapatinib, osimertinib, and CL-387,785 to penetrate the BBB have not been determined, lapatinib is expected to cross the BBB due to its low molecular weight and lipophilicity (Mansour et al., 2021b). However, studies of the use of BBB-penetrating EGFR inhibitors to treat AD remain scarce, and the mechanisms of BBB-permeable EGFR TKIs in AD remain to be elucidated. Several anti-cancer EGFR inhibitors that are candidates for AD therapy are described below, and the therapeutic effects, safety, and toxicity profiles of EGFR TKIs in cancer and AD are shown in Tables 1, 2; Figure 1B.

**TABLE 1:** The effects of EGFR inhibitors on cancer and AD.

EGFR Inhibitor	BBB Penetration	Effects in Cancer	Effects in AD	References
Gefitinib	0.0092 Brain Kpuu in WT rats	- First-generation EGFR TKI for lung cancer treatment	- Ameliorates Aβ-induced memory loss in APP/PS1 transgenic mice and a *Drosophila* AD model	Wang et al. (2012), Niu et al. (2014), Arrieta et al. (2020), Colclough et al. (2021), Dhamodharan et al. (2022)
			- Inhibits extracellular Aβ_40_/_42_ levels and reduces β-secretase (BACE-1) activity in APP-overexpressing N2a cells	
			- Improves cognition function in Swiss albino mice	
Erlotinib	0.084 Brain Kpuu in WT rats	- Suppresses tumor growth in the human endometrial adenocarcinoma cell line HEC-1A	- Significantly increases the performance index of Drosophila with Aβ_42_-induced memory loss	Wang et al. (2012), Nishimura et al. (2015), Colclough et al. (2021)
Afatinib	0.0046 Brain Kpuu in WT rats	- Second-generation EGFR-TKI with anti-inflammatory effects	- Prevents astrocyte activation	Chen et al. (2019a), Chen et al. (2019b), Vengoji et al. (2019), Colclough et al. (2021)
		- Inhibits the proliferation, migration, and invasion of hepatocellular carcinoma (HCC) cells		
		- Inhibits brain tumor formation by regulating EGFRvIII-cMet signaling when combined with temozolomide in glioblastoma cells	- Reduces proinflammatory cytokine levels and caspase-1 activation in CTXTNA2 cells	
Varlitinib	Crosses BBB	- FDA-approved EGFR/HER2 inhibitor	- Downregulates LPS-mediated neuroinflammatory responses and tau pathology in wild-type and tauoverexpressing PS19 mice	Babu et al. (2015), Liu et al. (2019), Dokduang et al. (2020), Kim et al. (2022)
		- Suppresses cell migration, invasion, and mammosphere formation in triple-negative breast cancer (TNBC) cells		
Lapatinib	Crosses BBB	- Dual TKI targeting EGFR and HER2	- Decreases Aβ_1_–_42_ and p-tau levels and ameliorates cognitive impairment in D-galactose/ovariectomized rats	Oakman et al. (2010), Matsumoto et al. (2018), Cihan (2019), Mansour et al. (2021b)
		- Antitumor effects in HER2-positive breast cancer cells		
Osimertinib	0.21 Brain Kpuu in WT rats	- Clinical activity against EGFR-mutant glioblastoma and non-small cell lung cancer (NSCLC)	- No specific studies of osimertinib as an AD therapy	Makhlin et al. (2019), Colclough et al. (2021), Gen et al. (2022)
CL-387,785	Expected to cross BBB	- Inhibits EGFR mutants more effectively than first/secondgeneration EGFR TKIs	- Decreases C99 and AICD levels in cellular, zebrafish, and mouse models of AD	Greulich et al. (2005), Kobayashi et al. (2005), Wang et al. (2017)
			- Rescues cognitive impairment in APP/ PS1 mice	

**TABLE 2:** Safety, toxicity profiles, and target cancers of EGFR inhibitors.

EGFR Inhibitor	Target group	Target cancers	Safety profile	Toxicity profile	References
Gefitinib	- ATP binding sites of EGFR	- NSCLC	- Treated with optimal biological dosage (250 mg/day)	- Rash	Noble and Faulds (1999), Van Zandwijk (2003), Jiang et al. (2005), Arrieta et al. (2020)
				- Diarrhea	
				- Xeroderma	
				- Pruritus	
Erlotinib	- ATP binding sites of EGFR	- NSCLC	- Tolerate total 1,600 mg weekly dosing for cancer patients	- Skin rash	Cohen et al., 2005 Smith (2005), Kiyohara et al. (2013), Carter and Tadi (2020)
				- Xeroderma	
				- Pruritus	
				- Paronychia	
Afatinib	- EGFR, HER2/ErbB2 and ErbB4	- NSCLC	- Increased up to a maximum dosage of 50 mg/day	- Skin deformity	Ingelheim (2016), Lai et al. (2017), Zhang et al. (2017), Tanaka et al. (2019)
				- Diarrhea	
				- Paronychia	
				- Oral mucositis	
				- Anorexia	
Varlitinib	- EGFR and HER2/ErbB2	- Gastric cancer	- Maximum tolerated dosage of 300 mg began twice-daily (BID)	- No toxicity observed in CCA-inoculated mouse	Hotte et al. (2009), Dokduang et al. (2020)
		- Pancreatic cancer			
		- Colorectal cancer			
		- Breast cancer			
Lapatinib	- EGFR and HER2/ErbB2	- Breast cancer	- Maximum tolerated dosage of 1,500 mg began twice-daily (BID)	- Diarrhea	Moy and Goss (2007), Oakman et al. (2010), Morikawa et al. (2019)
				- Rash	
Osimertinib	- Mutant-selective EGFR (exon 19 deletion EGFR)	- NSCLC	- Optimal toxic limit of 259 ng/mL	- Skin rash	Jiang and Zhou (2014), Makhlin et al. (2019), Agema et al. (2022)
				- Paronychia	
				- Acrodermatitis	
CL-387,785	- EGFR and mutant EGFR	- NSCLC	- Studies regarding toxicity not reported	- Studies regarding toxicity not reported	Greulich et al. (2005), Kobayashi et al. (2005), Wang et al. (2017)

### 5.1 Gefitinib

The EGFR/HER2 inhibitor Gefitinib is a first-generation EGFR TKI approved for lung cancer treatment (Arrieta et al., 2020). Gefitinib inhibits the binding of EGFR and adenosine triphosphate (ATP), thereby blocking EGFR autophosphorylation and downstream signaling cascades that control cell growth and trigger apoptosis (Jiang et al., 2005). Compared with erlotinib, gefitinib appears to be more effective and safer for treating NSCLC (Zhang et al., 2018).

Several studies have shown that gefitinib can ameliorate AD pathology. Gefitinib can penetrate the brain and thus positively affects non-EGFR targets that participate in AD pathology in E4FAD (APOE4-expressing) AD transgenic (Tg) mice (Thomas et al., 2016). A computational analysis indicated that gefitinib and the hydrophobic pocket of β-site APP cleaving enzyme (BACE) have complementary shapes and binding interactions, suggesting that gefitinib suppresses Aβ_40/42_ through BACE (Niu et al., 2014). In addition, gefitinib prevents memory loss in an Aβ_42_-overexpressing *Drosophila* model and rescues memory impairment in APP/PS1 Tg mice, a model of AD, indicating that EGFR inhibition can potentially improve cognitive function (Wang et al., 2012). In AD-induced Swiss albino mice, gefitinib attenuates hippocampal-dependent memory impairment as assessed by the Morris water maze (MWM) test and reduces acetylcholinesterase (AChE) activity (Dhamodharan et al., 2022). The ability of gefitinib to reduce Aβ-mediated AChE levels and attenuate cognitive impairments supports its potential as an AD treatment, but whether gefitinib directly affects other AD-associated factors (e.g., tau pathology) and its molecular mechanisms of action on AD pathology remain to be clarified.

### 5.2 Erlotinib

The EGFR-TKI erlotinib is an FDA-approved drug used to treat patients with NSCLC with mutations in the ATP-binding pocket of EGFR (Cohen et al., 2005; Smith, 2005; Nishimura et al., 2015; Lee et al., 2021b). Erlotinib suppresses the tumor growth of the human endometrial adenocarcinoma cell line HEC-1A, which expresses high levels of EGFR (Nishimura et al., 2015). Deep learning and machine learning algorithms predict that erlotinib is a BBB-permeable compound (Jang et al., 2022). Erlotinib blocks lipopolysaccharide (LPS)-induced nuclear factor kappa-light-chain-enhancer of activated B cells (NF-κB)-dependent cytokine production in C57BL/6J mice, implying that erlotinib modulates neuroinflammatory responses in the brain (De et al., 2015). However, due to their low BBB penetration, the effects of erlotinib and gefitinib on brain metastasis are neither significant nor persistent (Ahluwalia et al., 2018).

Importantly, erlotinib rescues memory deficits in APP/PS1 Tg mice as assessed by the MWM test, suggesting that erlotinib can modulate cognitive function (Wang et al., 2012). Although research has focused on the potential utility of erlotinib in treating AD, the effects of erlotinib on Aβ/tau pathology and its mechanisms of action in mouse models of AD require further study.

### 5.3 Afatinib

Afatinib is a second-generation EGFR-TKI with anti-inflammatory effects and is widely used to treat NSCLC (Chen et al., 2019b; Moosavi and Polineni, 2023). Afatinib also inhibits the migration, proliferation, and invasion of hepatocellular carcinoma (HCC) cells, implying anti-tumorigenic effects (Chen et al., 2019a). Treatment with a combination of afatinib and temozolomide suppresses brain tumor formation by inhibiting crosstalk between EGFRvIII, a constitutively active EGFR mutant, and the RTK cross-activation of tyrosine kinase receptor (cMet) (Vengoji et al., 2019). Interestingly, afatinib (1 or 10 nM) inhibits OGD-induced EGFR phosphorylation, astrocyte activation, and reduced proinflammatory cytokine levels in CTX-TNA2 cells (Chen et al., 2019b). These results suggest that afatinib has anti-inflammatory effects on OGD-induced neuroinflammation. However, whether afatinib regulates AD pathology and cognitive function in mouse models of AD is unknown. Although the direct effects of afatinib on AD pathology have not been comprehensively investigated, the anti-cancer and anti-inflammatory effects of afatinib indicate promising potential for repurposing as an AD therapy.

### 5.4 Varlitinib

Studies have examined the effects of varlitinib, an FDA-approved EGFR/HER2 inhibitor, on various cancers, including gastric, pancreatic, colorectal, and breast cancers (Dokduang et al., 2020). Varlitinib can penetrate the BBB and suppresses cell migration, invasion, and mammosphere formation through ERK/AKT signaling in triple-negative breast cancer (TNBC) cells (Babu et al., 2015; Liu et al., 2019). In addition, EGFR/HER2 inhibition by varlitinib has therapeutic effects on cholangiocarcinoma (CCA) (Dokduang et al., 2020). Whereas other EGFR inhibitors have side effects, varlitinib does not have significant toxicity in CCA-inoculated mice (Dokduang et al., 2020). Importantly, we recently demonstrated that varlitinib downregulates LPS-mediated neuroinflammation and tau pathology through dual specificity tyrosine phosphorylation regulated kinase 1A (DYRK1A), a tau kinase (Kim et al., 2022). In addition, we found that varlitinib significantly diminishes LPS-induced neuroinflammation in both BV2 microglial cells and primary astrocytes, suggesting that varlitinib has therapeutic effects on neuroinflammation and tau pathology (Kim et al., 2022). In contrast to its effects on cancer, the impact of varlitinib on AD pathology (including Aβ pathology and its mechanism of action) is poorly understood; thus, further studies are needed to evaluate the feasibility of using varlitinib for the treatment of AD.

### 5.5 Lapatinib

Like varlitinib, lapatinib is a dual TKI targeting both EGFR and HER2 and is currently used to treat cancer (Oakman et al., 2010). Lapatinib is a low-molecular-weight and lipophilic molecule and can penetrate the BBB (Mansour et al., 2021b). In HER2-positive breast cancer cells, a combination of lapatinib and capecitabine has synergistic anti-tumor effects (Matsumoto et al., 2018).

Lapatinib has been shown to ameliorate autoimmune encephalomyelitis, a functional disorder of the CNS (Akama-Garren et al., 2015). In addition, lapatinib has neuroprotective effects against neuronal ferroptosis, indicating the involvement of ferroptosis in AD pathologies (Jia et al., 2020; Chen et al., 2021). A study of the effects of lapatinib on cognitive function *in vivo* found that lapatinib rescues short/long-term recognition memory impairment by activating the PI3K/AKT/glycogen synthase kinase-3 beta (GSK-3β) pathway in D-galactose/ovariectomized (D-gal/OVX) rats (Mansour et al., 2021b). The same study demonstrated that lapatinib decreases Aβ_1-42_ and p-tau levels and suppresses HER2 expression in D-gal/OVX rats (Mansour et al., 2021b). Thus, inhibition of HER2 by lapatinib promotes autophagy and reduces Aβ_1–42_ and p-tau levels, consistent with the results of previous studies of the role of EGFR/HER2 in autophagy (Mansour et al., 2021a; Mansour et al., 2022). Overall, lapatinib is a promising candidate anti-cancer drug for repositioning as an AD therapeutic. However, further studies of the effects of lapatinib on AD pathophysiology are needed.

### 5.6 Osimertinib

Osimertinib is an irreversible EGFR inhibitor and a third-generation TKI with high brain penetration (Makhlin et al., 2019). Osimertinib targets EGFR T790, which is resistant to most first- and second-generation EGFR TKIs (Janjigian et al., 2014; Jiang and Zhou, 2014; Barbuti et al., 2019). Osimertinib is known for its clinical activities in glioblastoma and NSCLC (Makhlin et al., 2019; Gen et al., 2022). *In vitro*, osimertinib has higher affinity for EGFR L858R/T790M than for wild-type EGFR (Kuijper et al., 2015; Greig, 2016). Interestingly, a recent report indicated that osimertinib is also highly effective against CNS metastasis (Liam, 2019). Furthermore, Ge et al. (2017) observed a higher probability and frequency of EGFR mutations in NSCLC patients with brain metastasis than in NSCLC patients without brain metastasis. In addition, the incidence of brain metastasis is higher in NSCLC patients with mutated EGFR than in NSCLC patients with wild-type EGFR, implying a correlation between EGFR mutation and brain metastasis (Ge et al., 2017). Whether the potential effectiveness of osimertinib in the CNS extends to AD pathology is unknown.

The relationships between specific EGFR mutations (exon 19 deletions and exon 21 L858R) and AD have not been examined. Jolly et al. (2022) investigated the associations of the locations of mutations in EGF-like repeats (EGFr) with vascular cognitive impairment (VCI) in patients with cerebral autosomal dominant arteriopathy with subcortical infarcts and leukoencephalopathy (CADASIL), one of the most common forms of stroke and early-onset dementia, and found no significant relationship (Jolly et al., 2022). Although these results are not consistent with the mechanism of osimertinib, different results might be obtained for the relationships between EGFR mutations and ADs. Osimertinib may have utility as an EGFR inhibitor for treating AD pathology due to its high brain penetration, but the impact of osimertinib on AD has not been studied.

### 5.7 CL-387,785

CL-387,785 is an irreversible selective EGFR inhibitor designed to specifically inhibit EGFR autophosphorylation and tumor cell proliferation (Discafani et al., 1999). CL-387,785 inhibits not only wild-type EGFR but also EGFR T790M, which is resistant to EGFR TKIs such as erlotinib, gefitinib, and afatinib (Kobayashi et al., 2005; Yun et al., 2008; Mok et al., 2009; Rosell et al., 2012; Zhang et al., 2012; Chi et al., 2013; Sequist et al., 2013; Niu and Wu, 2014). Thus, CL-387,785 is expected to solve the cause of drug resistance. Although CL-387,785 is only approved for research purposes, several studies have indicated therapeutic effects of CL-387,785 on lung cancer. For instance, CL-387,785 inhibits colony formation by lung cancer cells expressing an EGFR missense or deletion mutant more effectively than gefitinib and erlotinib, suggesting that CL-387,785 may be a good therapeutic for lung cancer with exon 20 insertion mutations of EGFR (Greulich et al., 2005). In addition, CL-387,785 inhibits the proliferation and apoptosis of NSCLC H1975 cells expressing EGFR T790M, indicating that CL-387,785 can restrict the invasion and metastasis of NSCLC H1975 cells (Cai et al., 2023).

With respect to potential effects on AD, CL-387,785 significantly reduces C99-CTF (c-terminal fragment) and APP intracellular domain (AICD) levels in C99-YFP–overexpressing HEK293 cells and C99 CTF-expressing zebrafish (Wang et al., 2017). More importantly, CL-387,785 rescues spatial learning and memory and reduces Aβ levels in APP/PS1 Tg mice (Wang et al., 2017). CL-387,785 also reduces the LC3-II/LC3-I ratio, which is crucial for activating autophagy, promoting the clearance of Aβ_40_ and Aβ_42_, and improving memory (Wang et al., 2017). Although the effects of CL-387,785 on AD pathology (i.e., tau) and its mechanism of action require further investigation, CL-387,785 can be considered a potential EGFR TKI for both cancer and AD.

## Conclusion and future directions

Several recent studies have revealed associations of EGFR with cancer and AD; thus, regulating EGFR expression may be a strategy for treating both diseases. However, comprehensive studies of the roles of EGFR and EGFR inhibitors (TKIs) in cancer and AD are not available. In addition, although EGFR is a potential target for AD treatment, the effectiveness of major anti-cancer EGFR TKIs as AD therapeutics has received little attention. This review highlights the functional roles of EGFR and EGFR TKIs in cancer and AD. Specifically, EGFR upregulation induces various types of cancer and promotes Aβ pathology. EGFR inhibition has promising effects on both diseases, including inhibiting cancer cell migration and AD pathology (e.g., Aβ, neuroinflammation, and cognitive function). The literature and our findings suggest that anti-cancer drugs can be regarded as candidates for repurposing as AD treatments. However, the direct relationship between EGFR and AD, the effects of EGFR on tau pathology in mouse models of AD, and the mechanisms of action of EGFR in the brain are still unclear. Moreover, the effects of EGFR TKIs on AD pathology have not been well examined. Further studies are required to address these issues and may provide significant insights into cancer therapy and AD progression.
